# Supplementary material for: Coherence-controlled chaotic soliton bunch
Source: Nat Commun. 2024 Jul 21;15:6148. doi: 10.1038/s41467-024-50236-w (PMC11271477; doi:10.1038/s41467-024-50236-w)
Supplement: Supplementary file 1 — Supplementary Information [file 41467_2024_50236_MOESM1_ESM.pdf]

Supplementary Information for

Coherence-controlled chaotic soliton bunch

Ze-Xian Zhang[1,2], Min Luo[1,2], Jia-Hao Liu[1,2], Yi-Tao Yang[1,2], Ti-Jian Li[1,2], Meng Liu[1,2], Ai-Ping Luo[1,2], Wen-Cheng Xu[1,2], and Zhi-Chao Luo[1,2,*]

[1]Guangdong Provincial Key Laboratory of Nanophotonic Functional Materials and Devices, Guangdong Basic Research Center of Excellence for Structure and Fundamental Interactions of Matter, School of Information and Optoelectronic Science and Engineering, South China Normal University, Guangzhou, Guangdong 510006, China

[2]Guangzhou Key Laboratory for Special Fiber Photonic Devices and Applications, South China Normal University, Guangzhou, Guangdong 510006, China

*Correspondence should be addressed to Z.C.L. (zcluo@scnu.edu.cn).

Supplementary Note 1:

Formation mechanism for the oscillating tails of FOD-driven soliton

As is widely recognized, self-phase modulation (SPM) induces an intensity-dependent phase shift. When the pulse propagating in the optical fiber is assumed to be a Gaussian pulse, the nonlinear phase shift varies temporally and possesses a Gaussian profile as well, as depicted in Supplementary Fig. 1 with the blue dashed curve. Regarding the phase shift induced by fourth-order dispersion (FOD), it has been demonstrated that the phase shift profile has a quartic relationship with time, as plotted in Supplementary Fig. 1 with the red curve. It is worth noting that a critical condition for stable propagation of a soliton in the optical fiber is that the frequency chirps induced by SPM and fiber dispersion can be fully balanced. However, as observed in Supplementary Fig. 1, only the central part of the optical pulse can achieve a perfect balance between the phase shifts induced by SPM and FOD. For the two edges of the pulse, it is apparent that the phase shifts induced by SPM and FOD cannot be balanced. Therefore, if a Gaussian pulse is

input into an optical component with pure FOD, the energy of the optical pulse will flow from the center part to the two edges due to the imperfect counterbalance between the phase shifts induced by SPM and FOD. In this scenario, the oscillating tails can be observed with a soliton dominated by FOD and SPM.

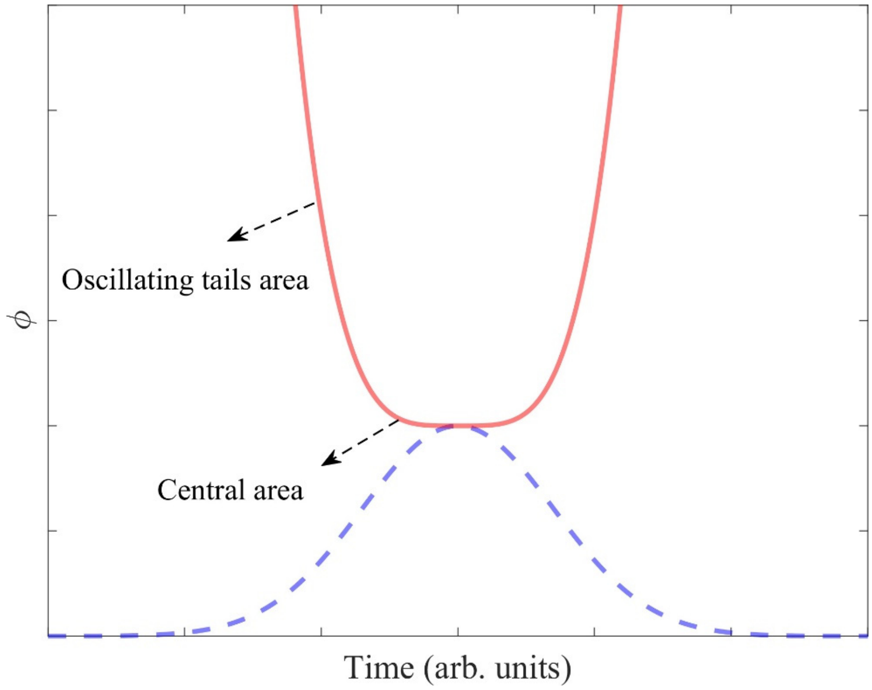

Supplementary Fig. 1 Phase profiles induced by SPM (blue curve) and FOD (red curve).

The formation of oscillating tails in the optical pulse leads to a variation in the pulse intensity profile. As mentioned above, the phase shift induced by SPM is intensity-dependent. Thus, the SPM-induced phase shift will change according to the variation in the intensity profile of the optical pulse (an optical pulse with oscillating tails). It has been demonstrated that the frequency chirp induced by SPM is linear and positive (up-chirp) over a large central region of the optical pulse. In this case, the SPM-induced chirp would be positive across both the main lobe of the optical pulse and the oscillating tails. On the other hand, the frequency chirp induced by FOD across both the main lobe of the optical pulse and the tails is negative (down-chirp), as shown in Supplementary Fig.2. Therefore, this provides a condition for the balance between the frequency chirps induced by SPM and FOD, both for the main lobe of the pulse and the oscillating tails. Combining this with the self-organization effect of an optical soliton, the stable propagation of the

optical soliton with the oscillating tails can be achieved.

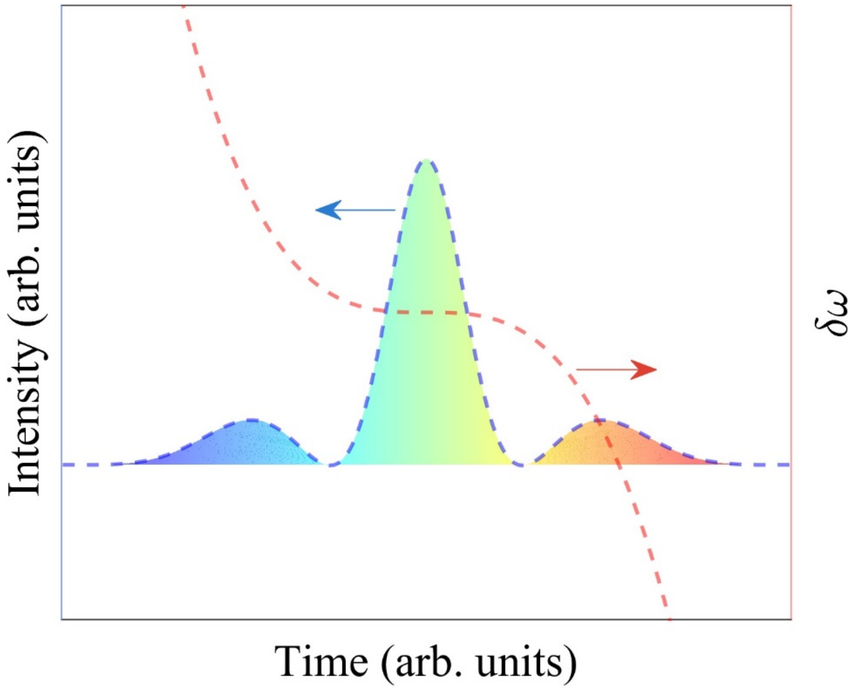

Supplementary Fig. 2 Intensity profile of optical soliton with oscillating tails (blue dashed curve) and FOD-induced chirp profile (red dashed curve).

Supplementary Note 2:

Controlling the intensity of oscillating pulse tails

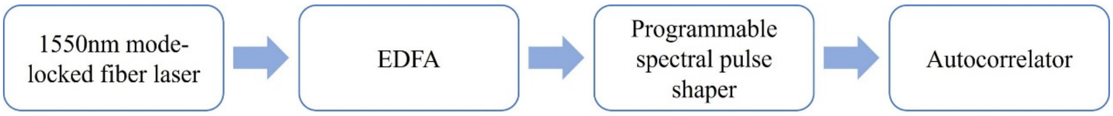

Supplementary Fig. 3 Setup for control of the oscillating tails.

An experimental platform for characterizing the control of oscillating tails is constructed, which is presented in Supplementary Fig. 3. Firstly, the optical pulse from a home-made ultrafast fiber laser at 1.55 μm waveband is injected into an erbium-doped fiber amplifier (EDFA) for the purpose of power scaling. In this case, the power of the optical pulse is sufficient after passing through a programmable spectral pulse shaper, ensuring that the intensity of the generated oscillating tails is strong enough to be measured by an autocorrelator. Note that the chirp of the mode-locked pulse induced by GVD is compensated to be near 0 and the FOD can

be flexibly adjusted by virtue of the spectral pulse shaper.

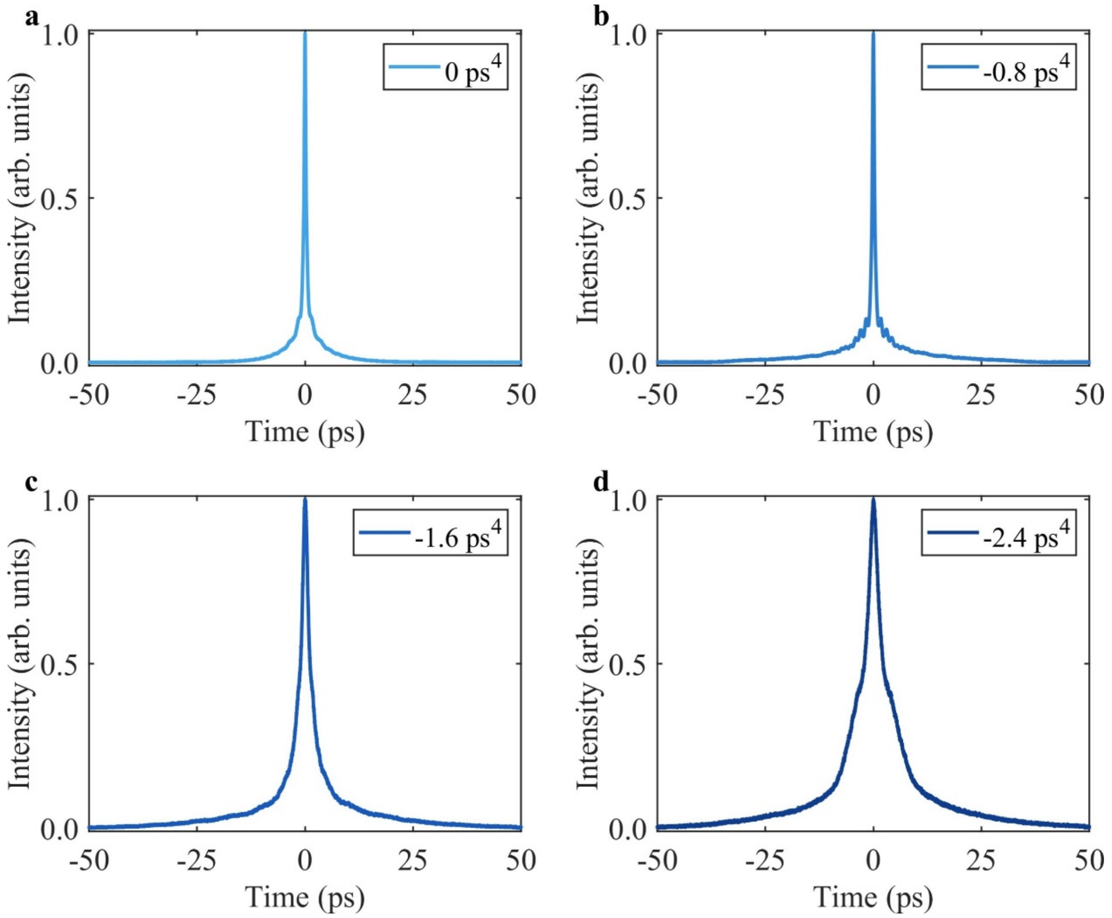

Supplementary Fig. 4 Experimental results of the autocorrelation trace with different amounts of FOD. a $0\ ps^4$, **b** $-0.8\ ps^4$, **c** $-1.6\ ps^4$, **d** $-2.4\ ps^4$.

As expected, by loading phase masks corresponding to different magnitudes of FOD into the programmable spectral pulse shaper, effective control over the oscillating tails of the mode-locked pulse can be achieved, as shown in Supplementary Fig. 4. It can be observed that, as the FOD amount experienced by the optical pulse increases, the pedestal of the autocorrelation trace becomes larger. The gradually strengthening pedestal in the autocorrelation trace reflects the increasing intensity of the oscillating pulse tails. To further validate the experiment, the numerical simulation of the experimental process was also conducted, with the results shown in Supplementary Fig. 5. The simulation results show that, as the amount of FOD increases, the temporal envelope exhibits larger oscillating tails, which is well consistent with the experimental findings.

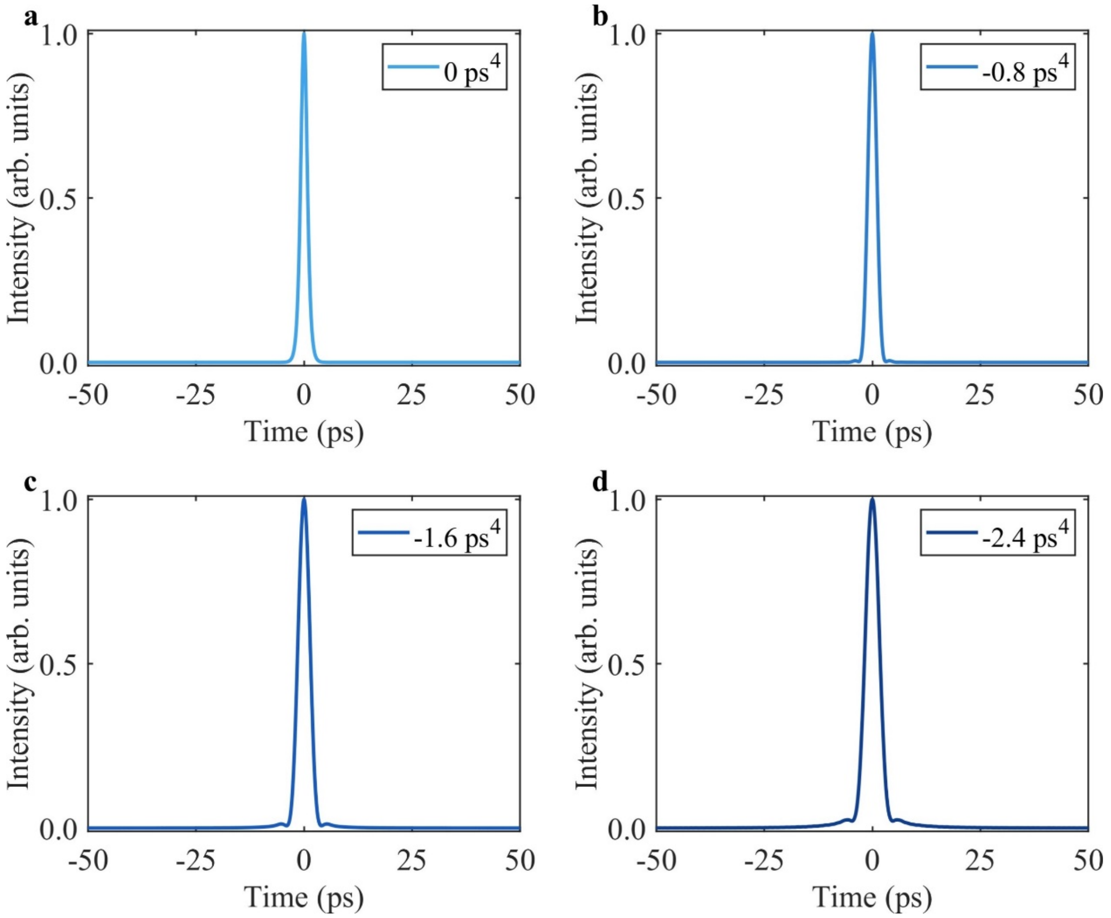

Supplementary Fig. 5 Simulation results of the pulse envelope with different amounts of FOD. a $0\ ps^4$, **b** $-0.8\ ps^4$, **c** $-1.6\ ps^4$, **d** $-2.4\ ps^4$.

Supplementary Note 3:

Experimentally measured features of chaotic soliton bunch in a GVD-dominated fiber laser

In order to compare the characteristics of chaotic pulse bunch in FOD- and group velocity dispersion (GVD)-dominated fiber lasers, we purposely remove the FOD generated by the spectral pulse shaper and record the laser performance of the chaotic pulse bunch in the fiber laser. The results are illustrated in Supplementary Fig. 6. As can be observed from Supplementary Fig. 6a, the spectrum of chaotic pulse bunch from the fiber laser dominated by GVD is smooth and the Kelly sidebands are absent in this case, similar to early reports[1]. This result suggests the complete destruction of the interference between mode-locked soliton and dispersive waves. The autocorrelation trace in Supplementary Fig. 6b clearly

demonstrates that the fiber laser is operating in the regime of chaotic soliton bunch, featuring a narrow coherent peak with a broad shoulder.

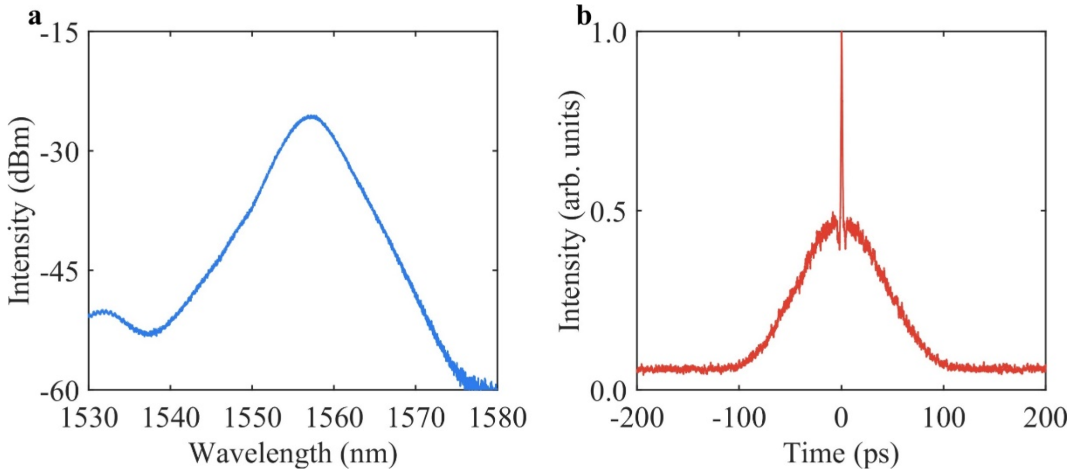

Supplementary Fig. 6 Experimental results of chaotic pulse bunch emitted by a GVD-dominated fiber laser. a Optical spectrum recorded with an OSA. **b** Autocorrelation trace.

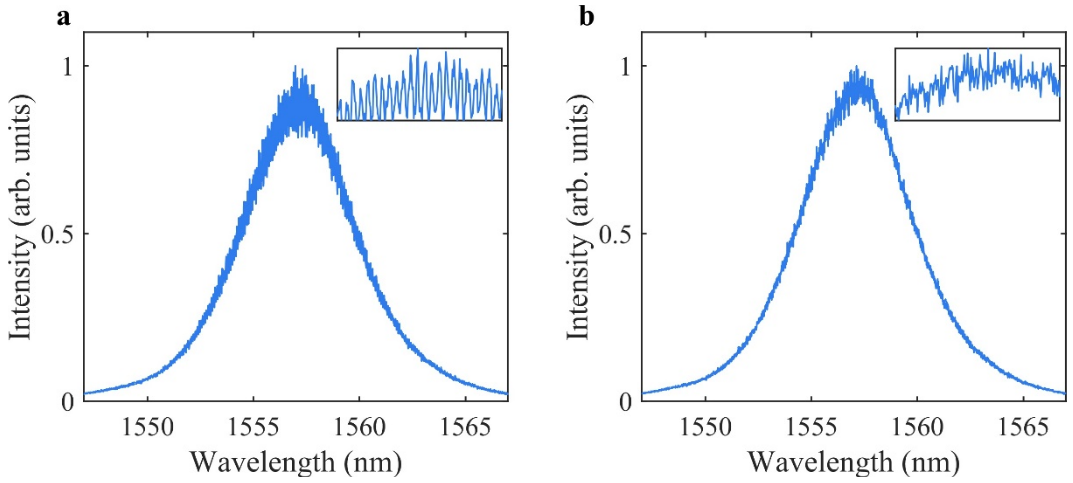

Supplementary Fig. 7 Coherence of the chaotic soliton bunch in a GVD-dominated fiber laser. a Spectral interference pattern after passing a M-Z interferometer. **b** Spectrum without passing a M-Z interferometer. Inset: zoom-in of the spectral patterns.

To further reveal the coherence property, the phase coherence of the chaotic soliton bunch in the GVD-dominated fiber laser is also characterized by using a Mach-Zehnder interferometer, as displayed in Supplementary Fig. 7a. The relatively weak interference fringes can be observed on the mode-locked spectrum (inset of Supplementary Fig. 7a). Here, we should note that the spectral peaks can

also be observed when we remove the Mach-Zehnder interferometer (Supplementary Fig. 7b). Therefore, it can be concluded that the coherence of the chaotic soliton bunch emitted from the GVD-dominated fiber laser is relatively low[1].

Supplementary Note 4:

Autocorrelation traces of chaotic soliton bunches with two FOD values

Supplementary Fig. 8 shows the experimentally measured autocorrelation traces of the chaotic soliton bunch with different FOD amounts generated by spectral pulse shaper. The autocorrelation traces always exhibit a coherent narrow peak sitting on a large pedestal when the FOD are set to $\beta_4 = -1\ \mathrm{ps^4\ km^{-1}}$ (Supplementary Fig. 8a) and $\beta_4 = -10\ \mathrm{ps^4\ km^{-1}}$ (Supplementary Fig. 8b). These results indicate that the fiber laser maintains the operation regime of chaotic soliton bunch for both FOD values.

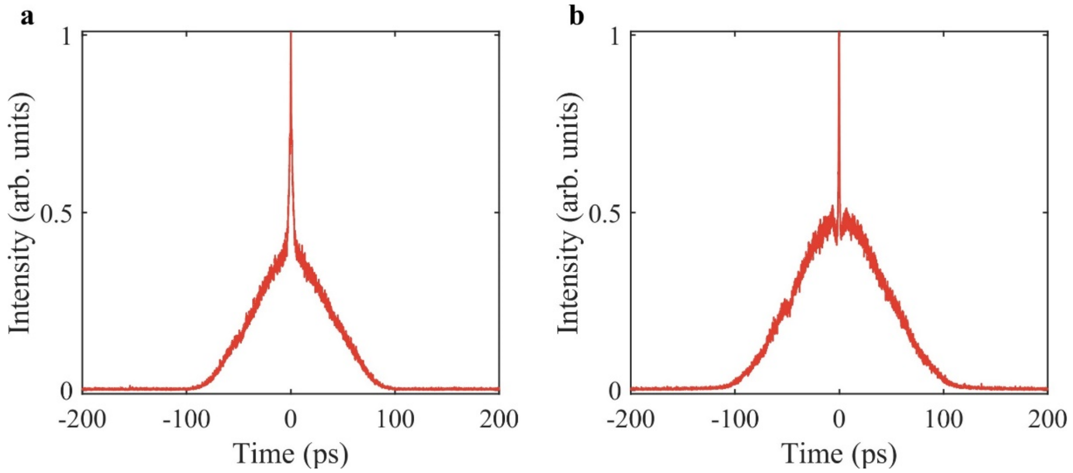

Supplementary Fig. 8 Autocorrelation traces of the chaotic soliton bunch with two different values of FOD. a $\beta_4 = -1\ \mathrm{ps^4\ km^{-1}}$. **b** $\beta_4 = -10\ \mathrm{ps^4\ km^{-1}}$.

Supplementary Note 5:

Variation of quartic dispersion length

As is well known before, the role of GVD on pulse shaping can be intuitively reflected by the quadratic dispersion length, which is defined by $L_{\mathrm{GVD}} = T_0^2/|\beta_2|$

(T_0 is pulse width). Similar to that of GVD, the role of FOD on pulse shaping can also be qualitatively reflected by the quartic dispersion length[2,3], which is defined as $L_{\mathrm{FOD}} = T_0^4/|\beta_4|$. Meanwhile, it is found that the pulse duration of the chaotic solitons within the bunch do not change much during the process of FOD adjustment, which exhibit an average pulse duration of ~1 ps. Then the dispersion length with a varying FOD can be calculated, as shown in Supplementary Fig. 9. It is observed that the evolution trend of the L_{FOD} with respect to $|\beta_4|$ is highly consistent with that of coherence and energy fluctuation of the chaotic soliton bunch. The L_{FOD} is reaching 1000 m when the β_4 is set to be $-1\ \mathrm{ps^4\ km^{-1}}$. Considering the cavity length is 30.05 m, the effect of FOD on pulse shaping is rather weak, that is, the coherence of the chaotic soliton bunch is relatively low, corresponding to a 0.27 fringe visibility of spectral interference pattern. When the L_{FOD} decreases to 100 m when β_4 is set to $-10\ \mathrm{ps^4\ km^{-1}}$, the FOD has a significant impact on the shaping of chaotic soliton bunch, with a fringe visibility of 0.58. In fact, the evolution curve of dispersion length with the FOD also further verifies that the coherence of chaotic soliton bunch relies on the role of FOD, as the evolution curves of the dispersion length and the coherence of chaotic soliton bunch with an increasing FOD exhibit the same trends.

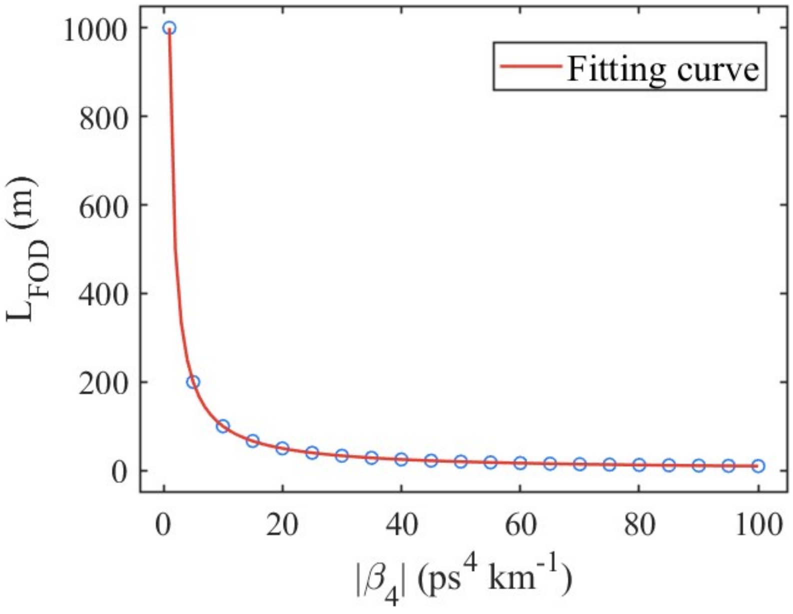

Supplementary Fig. 9 Quartic dispersion length with a varying $|\beta_4|$.

Supplementary Note 6:

Coherence control of chaotic soliton bunch without compensating GVD

Both the experiments and simulations for the coherence control of chaotic soliton bunch under the condition of GVD cancellation are demonstrated to enhance the effect of FOD on pulse shaping in our laser cavity. Here, the coherence control of chaotic soliton bunch without purposely compensating GVD ($\beta_2 = -4.2642\ \mathrm{ps^2\ km^{-1}}$) by varying the FOD in both experiments and numerical simulations are shown in Supplementary Fig. 10. It can be found that the conclusion of the coherence control of chaotic soliton bunch is still well established with FOD variation. In the experiment, the visibility of the interference fringe increases from 0.54 to 0.73 when the FOD increases from $-5\ \mathrm{ps^4\ km^{-1}}$ to $-100\ \mathrm{ps^4\ km^{-1}}$ correspondingly, showing an evident trend from low to high coherence, as plotted in Supplementary Fig. 10a. Meanwhile, the RMS of spectral peak variation is decreasing, as shown in Supplementary Fig. 10b. Again, the simulation results are well consistent with the experimental ones, as demonstrated in Supplementary Fig. 10c and Supplementary Fig. 10d. That is to say, a certain amount of GVD introduced in the laser cavity does not break the conclusion of coherence control of chaotic soliton bunch by adjusting the FOD. Nevertheless, owing to the existence of GVD, the achievable controlling range of the bunch-to-bunch coherence is smaller than in the case of GVD cancellation, because the pulse dynamics of the fiber laser are dominated by GVD when the FOD is small initially. In this case, the effect of GVD will have a significant impact on the mode-locked state. Hence, the GVD is compensated to be 0 to enhance the effect of FOD on the coherence-controlled property of chaotic soliton bunch.

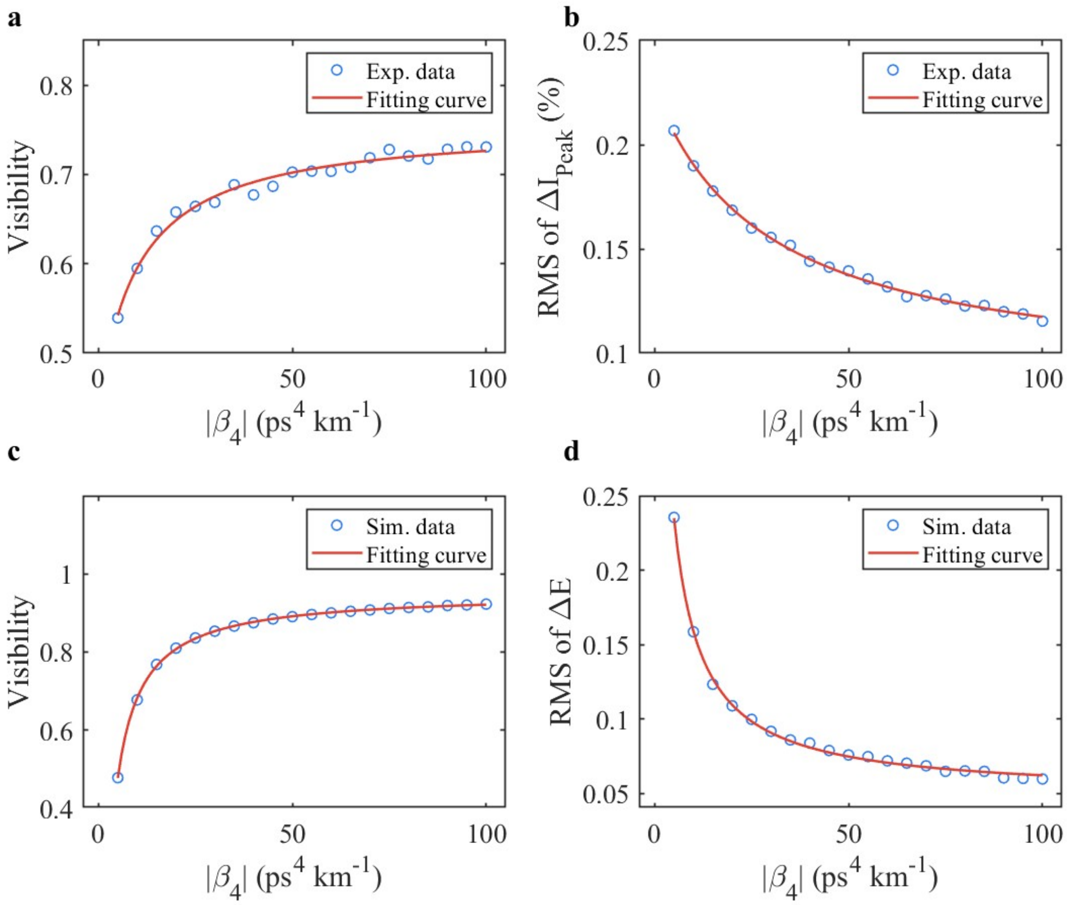

Supplementary Fig. 10 Fringe visibility of spectral interference pattern and RMS of intensity fluctuation of chaotic soliton bunch with a certain amount of GVD and a varying $|\boldsymbol{\beta_4}|$. a,b experimental results; **c,d** numerical simulations.

Supplementary Note 7:

Simulation results of FOD-driven single soliton operation

In addition to the chaotic soliton bunch regime, the stable single soliton supported by FOD, namely pure-quartic soliton (PQS) operation can also be achieved with $E_s = 2$ pJ and $\beta_4 = -20$ ps^4 km^{-1}. Supplementary Fig. 11a and Supplementary Fig. 11b are the temporal and spectral profiles of the stable single PQS, respectively. They exhibit the typical characteristics of PQS, such as temporal oscillating tails and spectral sidebands that are related to FOD[2,4]. Moreover, the evolutions in the time and spectral domain both show the good stability of the mode-locked PQS, as demonstrated in Supplementary Fig. 11c and Supplementary Fig. 11d.

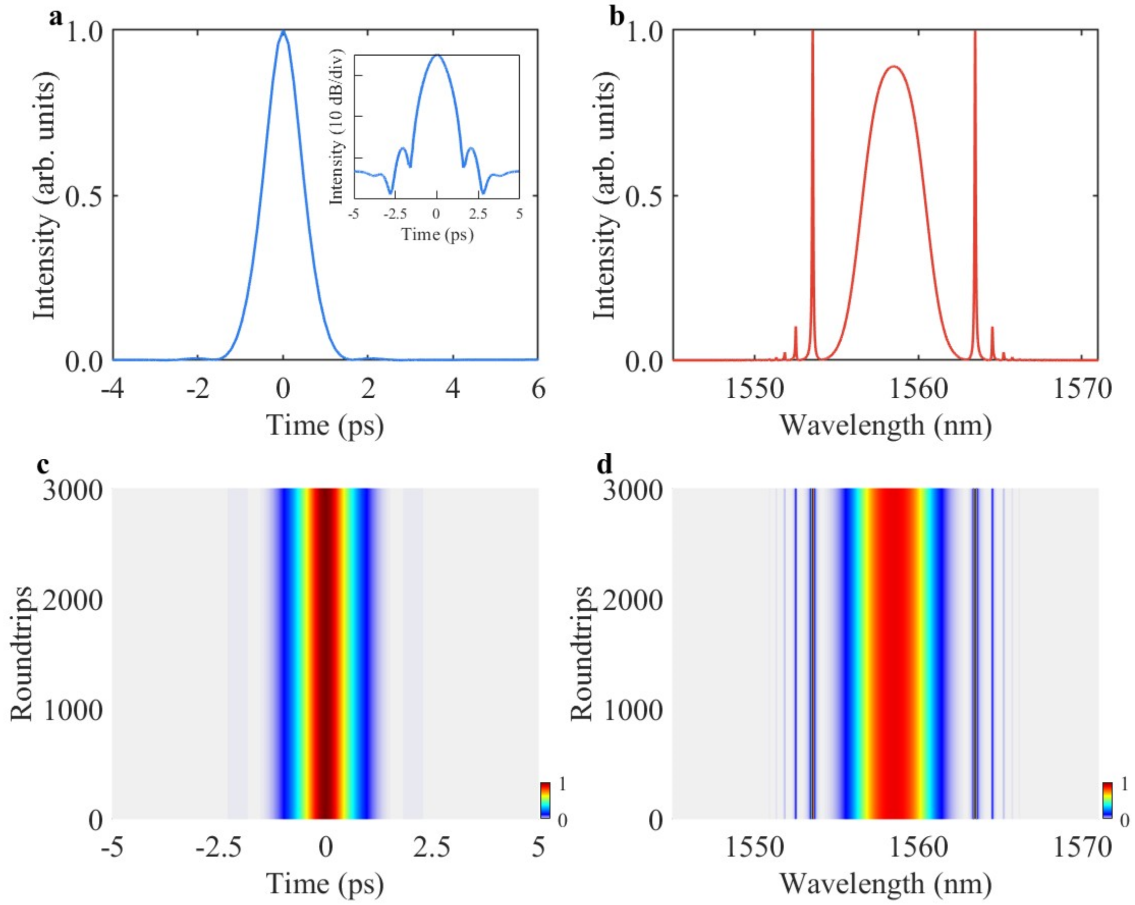

Supplementary Fig. 11 Simulation results of the stable single soliton operation dominated by FOD. a Temporal and **b** spectral profiles of stable single soliton. Evolution in the **c** time and **d** spectral domain.

Supplementary Note 8:

Simulation results of GVD-driven chaotic soliton bunch

The energy fluctuation of the chaotic soliton bunch with $\beta_4 = -20\ \mathrm{ps^4\ km^{-1}}$ is presented in Supplementary Fig. 12a, which corresponds to the RMS of ΔE = 0.1335 %. As demonstrated in Supplementary Fig. 12b, the energy fluctuation of the chaotic pulse bunch without FOD is larger than that of the chaotic soliton bunch with FOD, and the RMS of ΔE is calculated to be 0.3436 %. Furthermore, the spectral evolution, as well as the averaged spectrum, exhibits the feature of the disappearance of the Kelly sidebands, as shown in Supplementary Fig. 12c. From Supplementary Fig. 12d, the sub-pulses in the conventional chaotic pulse bunch undergo a severe splitting and annihilation process, such that we cannot track any

of them, which is different from the case of the chaotic soliton bunch driven by FOD shown in Figure 4b of the manuscript. Briefly, we can claim that the lifetimes of sub-pulses in the conventional chaotic pulse bunch are much shorter than those of the FOD-driven chaotic soliton bunch. In turn, this result further reveals the reason for a lower energy fluctuation of the FOD-driven chaotic soliton bunch.

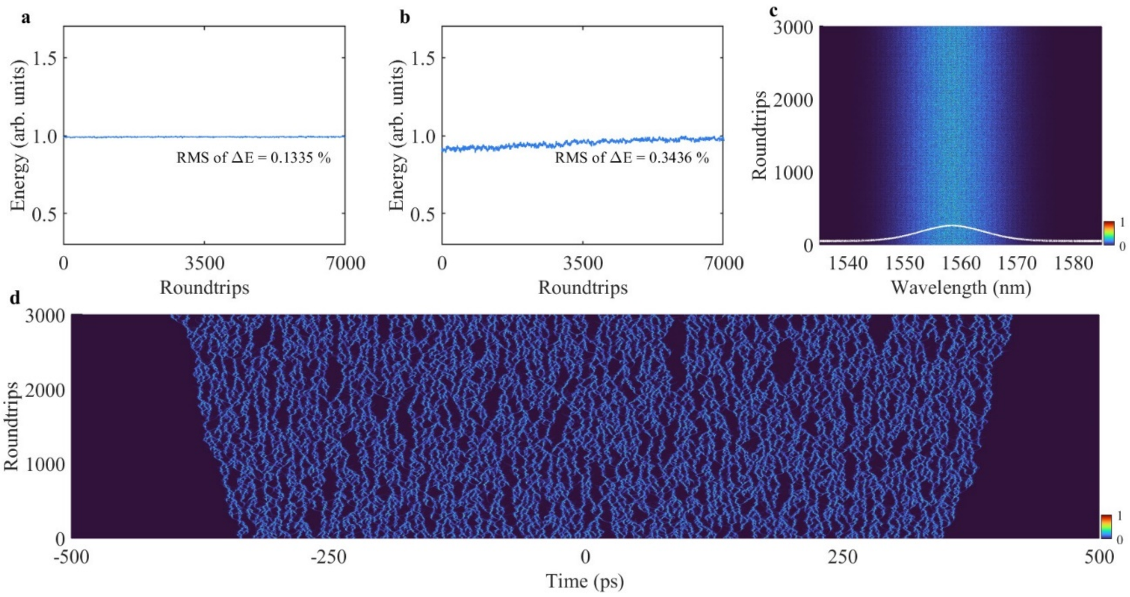

Supplementary Fig. 12 Simulated characteristics of chaotic soliton bunch in a GVD-dominated fiber laser. RMS of ΔE under the condition with **a** FOD and **b** without FOD. **c,d** Spectral and temporal evolution of chaotic soliton bunch generated from a GVD-dominated fiber laser. White curve: averaged spectrum over 3000 roundtrips.

Supplementary Note 9:

Experimental manipulation of optical rogue waves

The optical rogue waves can be generated by chaotic soliton collisions in a temporally localized bunch. Supplementary Figs. 13a-13d display the statistical distribution histograms of the spectral peak fluctuations over 10000 events under different FODs. It can be observed that with the increasing FOD, the significant wave height (SWH) also increases. Note that the criterion for identifying the optical rogue wave generation in this work is that the highest amplitude of waves is larger than twice the SWH, which is a widely adopted criterion in investigating

rogue waves according to previous reports[5]. This variation indicates that by adjusting the FOD, not only the coherence of chaotic soliton bunch can be controlled, but also the rogue waves can be manipulated.

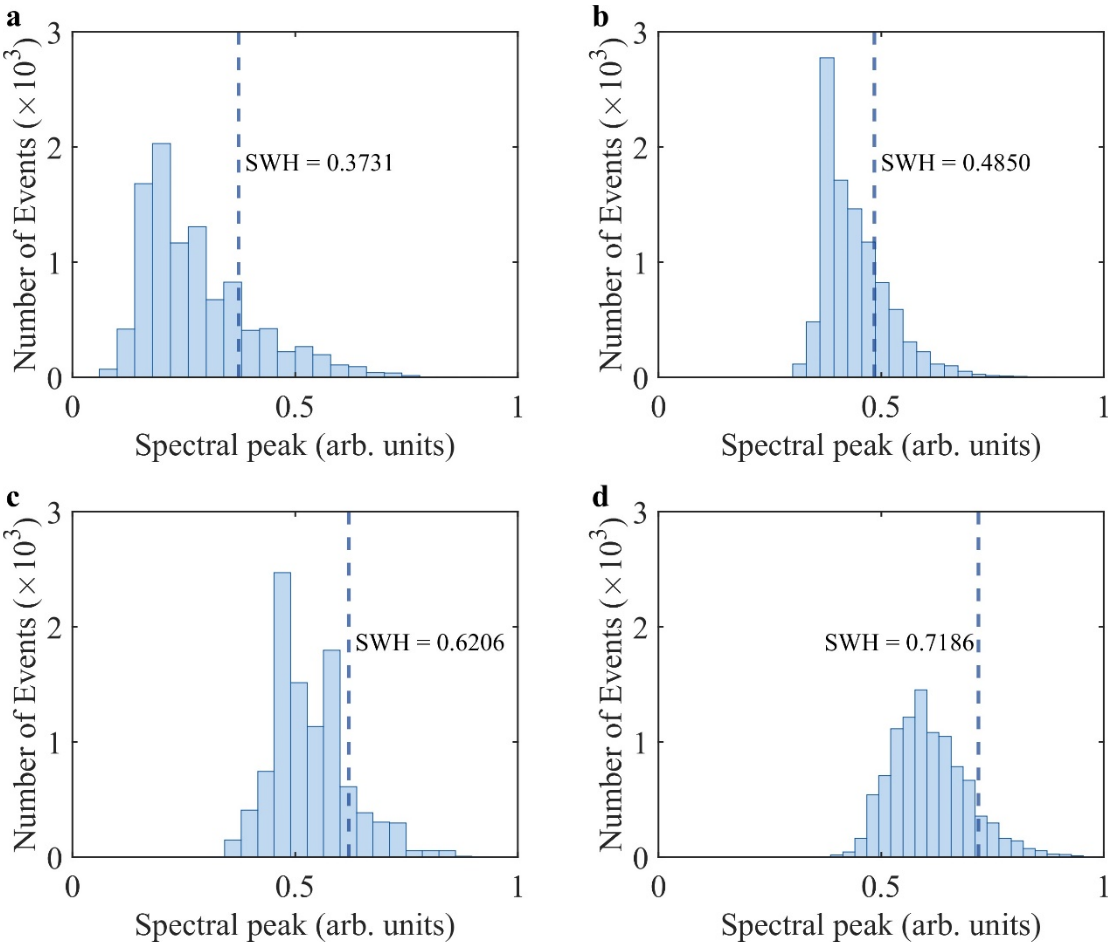

Supplementary Fig. 13 Manipulation of optical rogue waves. Statistical distribution histograms of the spectral peak fluctuations under a FOD of **a** $\beta_4 = -1$ ps^4 km^{-1}, **b** $\beta_4 = -5$ ps^4 km^{-1}, **c** $\beta_4 = -20$ ps^4 km^{-1}, and **d** $\beta_4 = -100$ ps^4 km^{-1}.

Supplementary References

1. Runge, A. F. J., Aguergaray, C., Broderick, N. G. R. & Erkintalo, M. Coherence and shot-to-shot spectral fluctuations in noise-like ultrafast fiber lasers. Opt. Lett. 38, 4327-4330 (2013).
2. Tam, K. K. K., Alexander, T. J., Blanco-Redondo, A. & de Sterke, C. M. Stationary and dynamical properties of pure-quartic solitons. Opt. Lett. 44, 3306-3309 (2019).
3. Blanco-Redondo, A. et al. Pure-quartic solitons. Nat. commun. **7**, 10427 (2016).
4. Runge, A. F. J., Hudson, D. D., Tam, K. K. K., de Sterke, C. M. & Blanco-Redondo, A. The pure-quartic soliton laser. Nat. Photonics 14, 492-497 (2020).
5. Lecaplain, C., Grelu, P., Soto-Crespo, J. M. & Akhmediev, N. Dissipative rogue waves generated by chaotic pulse bunching in a mode-locked laser. Phys. Rev. Lett. 108, 233901 (2012).
